# New Insights Into the Golgi Stacking Proteins

**DOI:** 10.3389/fcell.2019.00131

**Published:** 2019-07-16

**Authors:** Erpan Ahat, Jie Li, Yanzhuang Wang

**Affiliations:** ^1^Department of Molecular, Cellular and Developmental Biology, University of Michigan, Ann Arbor, MI, United States; ^2^Department of Neurology, University of Michigan School of Medicine, Ann Arbor, MI, United States

**Keywords:** Golgi, stacking, GRASP65, GRASP55, O-GlcNAcylation, autophagy, unconventional secretion

## Abstract

The Golgi stacking proteins, GRASP55 and GRASP65, are best known for their roles in Golgi structure formation. These peripheral Golgi proteins form *trans*-oligomers that hold the flat cisternal membranes into stacks. Depletion of both GRASP proteins in cells disrupts the Golgi stack structure, increases protein trafficking, but impairs accurate glycosylation, and sorting. Golgi unstacking by GRASPs depletion also reduces cell adhesion and migration in an integrin-dependent manner. In addition to Golgi structure formation and regulation of cellular activities, GRASPs, in particular GRASP55, have recently drawn attention in their roles in autophagy, and unconventional secretion. In autophagy, GRASP55 senses the energy level by O-GlcNAcylation, which regulates GRASP55 translocation from the Golgi to the autophagosome-lysosome interface, where it interacts with LC3 and LAMP2 to facilitate autophagosome-lysosome fusion. This newly discovered function of GRASP55 in autophagy may help explain its role in the stress-induced, autophagosome-dependent unconventional secretion. In this review, we summarize the emerging functions of the GRASP proteins, focusing on their roles in cell adhesion and migration, autophagy, unconventional secretion, as well as on novel GRASP-interacting proteins.

## Introduction

The Golgi apparatus is an essential membrane-bound organelle in the cell that functions as a “*post station*” in the secretory pathway (Klute et al., 2011). In mammalian cells, Golgi membranes are organized as stacks of multiple flat cisternae, which are further linked into a ribbon-like structure located in the perinuclear region (Klumperman, 2011). The Golgi functions as a protein modification and sorting center in the secretory pathway, with different modification enzymes residing in different subcompartments, including *cis*-Golgi network (CGN), *cis*-, *medial-*, and *trans*-cisternae, and *trans*-Golgi network (TGN) (Goldfischer, 1982). The Golgi receives newly synthesized proteins and lipids from the endoplasmic reticulum (ER), sequentially modifies, and dispatches them to distinct destinations by protein sorting at the TGN (Marsh and Howell, 2002; Brandizzi and Barlowe, 2013).

**FIGURE 1:**
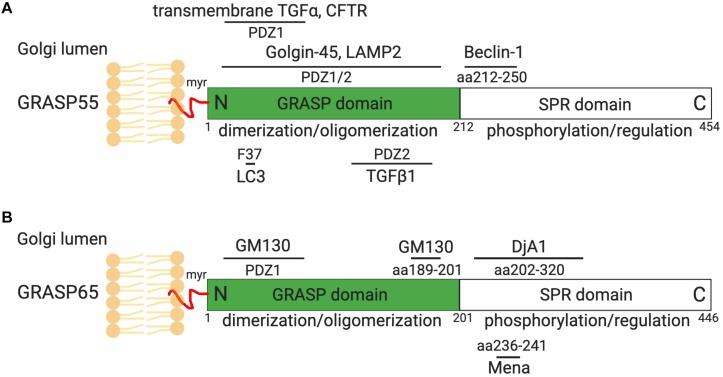
GRASP55 **(A)** and GRASP65 **(B)** domain structures and interacting proteins. GRASP55 and GRASP65 are peripheral membrane proteins that are attached to the Golgi membranes via myristoylation (myr). Both GRASPs share similar domain structures, with a conserved GRASP domain at the N-terminus consisted of PDZ1 and PDZ2 subdomains, and a C-terminal serine/proline-rich (SPR) domain. The GRASP domain forms dimers and *trans*-oligomers that tether adjacent membranes. The SPR domain contains multiple phosphorylation sites whose phosphorylation in mitosis impairs GRASP oligomerization and leads to Golgi fragmentation (Wang et al., 2003, 2005; Tang et al., 2012). GRASP55 and GRASP65 interacts with Golgin-45 and GM130, respectively, which are essential for their roles in Golgi structure formation and function (Barr et al., 1997; Short et al., 2001). GRASP55 also regulates autophagy by interacting with Beclin-1, LC3 and LAMP2, and controls CFTR, TGFα (pro form) and TGFβ1 secretion (Kuo et al., 2000; Gee et al., 2011; Nüchel et al., 2018; Zhang et al., 2018, 2019). GRASP65 interacts with Mena and Dja1, which are essential for Golgi structure formation (Tang et al., 2016; Li et al., 2019b). More GRASP-interacting proteins can be found on Table 1, only those with known binding sites on GRASPs are shown here. Indicated sites are based on rat GRASP sequences.

**FIGURE 2:**
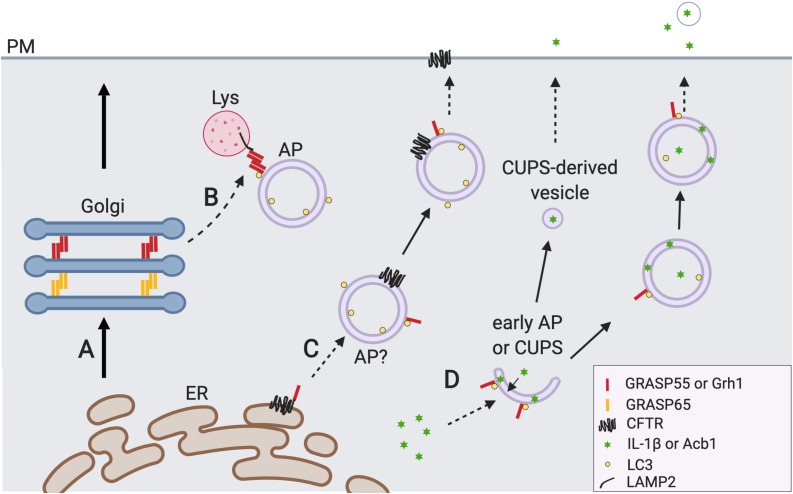
GRASP55 regulates autophagy and unconventional secretion. **(A)** The conventional ER-Golgi-plasma membrane (PM) secretory pathway. **(B)** GRASP55 facilitates autophagosome (AP)-lysosome (Lys) fusion. Upon glucose starvation or amino acid starvation, GRASP55 is translocated to the AP-Lys interface to promote autolysosome formation by bridging LC3 and LAMP2, and by facilitating the UVRAG PI3K complex formation (not shown) (Zhang et al., 2018, 2019). Undigested contents in the autolysosomes and lysosomes may be secreted by lysosome exocytosis (not shown) (Reddy et al., 2001; Samie and Xu, 2014). **(C)** GRASP55 is required for CFTR trafficking that bypasses the Golgi. Mutant CFTR is transported to the PM in a Golgi-independent manner under ER stress and inhibition of the conventional secretory pathway. During this process phosphorylated and monomerized GRASP55 binds CFTR at the ER membrane and regulates its sequestration (Kim et al., 2016). So far no evidence has been provided that CFTR is indeed localized on the outer membrane of AP, and how CFTR is translocated from ER to AP remains elusive. GRASP55 may also regulate this pathway by facilitating direct fusion of CFTR containing APs with PM or Lys via *trans*-oligomer formation (not reported). **(D)** GRASP55 is involved in unconventional secretion of leaderless cytosolic proteins (i.e., Acb1 and IL-1β). IL-1β could be secreted via secretory autophagosomes which is GRASP55 dependent (Zhang et al., 2015). The function of GRASP55 here is unclear but GRASP55 may serve as an ER stress sensor or UPR regulator in this process (Chiritoiu et al., 2019). Yeast protein Acb1 secretion requires the formation of a new type of Grh1-positive membrane compartment called CUPS (compartment for unconventional protein secretion) whose identity is largely unknown (Bruns et al., 2011). CUPS derived vesicles (saccules) fuse with plasma membrane and releases Acb1 to outside of the cell (Curwin et al., 2016).

The highly ordered Golgi stack structure, which is the functional unit of the Golgi, is believed to facilitate sequential protein modification, and processing in mammals. Using *in vitro* assays mimicking Golgi disassembly and reassembly that occur during the cell cycle, two Golgi peripheral membrane proteins, GRASP65 and GRASP55 (Golgi ReAssembly and Stacking Protein), were identified as Golgi stacking factors (Barr et al., 1997; Shorter et al., 1999; Wang et al., 2003). Both GRASPs were further characterized and confirmed to control Golgi stacking and ribbon linking *in vivo* (Wang et al., 2003; Puthenveedu et al., 2006; Feinstein and Linstedt, 2008; Xiang and Wang, 2010). GRASP65 is mainly targeted to *cis*-Golgi, whereas GRASP55 localizes to *medial*- and *trans*-cisternae. GRASP65 and GRASP55 have similar domain structures. The conserved GRASP domain at the N-terminus contains a membrane anchor and can form dimers and *trans*-oligomers. The more divergent serine proline-rich (SPR) domain at the C-terminus contains multiple phosphorylation sites, whose phosphorylation inhibits GRASP oligomerization in mitosis (Wang et al., 2005; Vielemeyer et al., 2009; Tang et al., 2012) and perhaps also in stress and pathological conditions (Figures 1, 2A; Joshi et al., 2014, 2015; Joshi and Wang, 2015). In coordination with GRASPs, GRASPs interacting proteins, including the GRASP65 binding partner GM130, and GRASP55 binding protein Golgin-45, may also be involved in Golgi structure formation (Lee et al., 2014).

**TABLE 1:** GRASP interacting proteins and functions.

**Names**	**Golgi localization**	**Interaction proteins**	**Functions**
GRASP55/GORASP2	*Medial/Trans*	– Golgin-45 (Short et al., 2001; Zhao et al., 2017) – p24 (Barr et al., 2001) – TGF-α (Kuo et al., 2000), CD8a and Frizzled 4 (D’Angelo et al., 2009) – LC3, LAMP2 (Zhang et al., 2018), Beclin-1, UVRAG (Zhang et al., 2019) – CFTR (Gee et al., 2011), TGFβ1 (Nüchel et al., 2018) – JAM (Cartier-Michaud et al., 2017)	Golgi stacking (Shorter et al., 1999; Short et al., 2001; Xiang and Wang, 2010; Bekier et al., 2017; Zhao et al., 2017) p24 cargo receptor retention (Barr et al., 2001) Transport of specific cargo (Kuo et al., 2000; D’Angelo et al., 2009) Autophagosome-lysosome fusion (Zhang and Wang, 2018a,b; Zhang et al., 2018) Unconventional secretion (Gee et al., 2011; Rabouille, 2017; Nüchel et al., 2018) Spermatogenesis (Cartier-Michaud et al., 2017)
GRASP65/GORASP1	*Cis*	– GM130 (Barr et al., 1998), DjA1 (Li et al., 2019b) – Mena (Tang et al., 2016) – p24 (Barr et al., 2001) – CD8a, Frizzled 4 (D’Angelo et al., 2009) – Bcl-X_L_ (Cheng et al., 2010), caspase-3 (Lane et al., 2002)	Golgi stacking (Barr et al., 1997; Xiang and Wang, 2010; Bekier et al., 2017; Li et al., 2019b) Golgi ribbon formation (Tang et al., 2016) p24 cargo receptor retention (Barr et al., 2001) Transport of specific cargo (D’Angelo et al., 2009) Apoptosis (Lane et al., 2002; Cheng et al., 2010)

Since depletion or inhibition of GRASP55 and GRASP65 impairs Golgi structure formation, these proteins have been recently used as tools to disrupt the Golgi structure and thereby determine the functional consequence of Golgi structural disruption. In addition, a number of novel GRASP-interacting proteins have been identified and GRASPs have been linked to autophagy, unconventional secretion and other cellular activities, such as cell adhesion, migration, and growth. In this review, we attempt to summarize these new discoveries on GRASP functions and discuss the potential links between these new findings.

## Grasp55 and Grasp65 as Tools to Probe the Biological Significance of Golgi Structure Formation

The role of GRASPs in Golgi stack formation and the impact of GRASP depletion or inhibition on Golgi functions have been explored using a number of experimental approaches. Inhibition of GRASP65 by microinjecting inhibitory antibodies, knocking down (KD) GRASPs by siRNA, or knocking out (KO) GRASPs by CRISPR/Cas9, all significantly impair Golgi stack formation (Wang et al., 2003; Tang et al., 2010; Bekier et al., 2017). Research from the Rothman and Wang labs demonstrated that depletion of GRASPs, which results in Golgi fragmentation, increases the trafficking of selected cargo molecules, including vesicular stomatitis virus G-protein (VSVG), α5 integrin, and CD8 (Wang et al., 2008; Xiang et al., 2013; Lee et al., 2014). A plausible explanation for this result is that Golgi unstacking increases the membrane surface for vesicle formation and thus accelerates protein trafficking (Wang et al., 2008; Zhang and Wang, 2015; Bekier et al., 2017; Ahat et al., 2019). Need to mention, in contrast to the results described above, D’Angelo et al. (2009) reported that GRASP55/65 bind the C-terminal hydrophobic tail of specific transmembrane proteins such as CD8, and this interaction is required for CD8 trafficking through the Golgi stack. These controversial results could be caused by the different constructs used in the studies; the first two labs used full length CD8 (or a fusion protein with full length CD8), and while the last one used a VSVG-CD8α (C-terminal tail) chimera. Other factors, such as the knockdown efficiency, may also be involved. Considering that there is currently no other way to disrupt Golgi stacking other than manipulating GRASPs, this subject requires further investigation.

Glycomic analysis by mass spectrometry showed that GRASP depletion, especially GRASP55 single depletion or GRASP55/65 double-depletion, results in a reduction in the overall glycan abundance, complexity, and glycoprotein composition at the plasma membrane. Interestingly, GRASP depletion-mediated Golgi unstacking also causes mis-sorting of lysosome enzymes such as cathepsin D (Xiang et al., 2013; Zhang et al., 2015). Therefore, it has been proposed that cisternal stacking impedes the intra-Golgi trafficking speed by reducing the accessibility of coat proteins to Golgi membranes, which ensures accurate glycosylation, and sorting (Zhang and Wang, 2015, 2016; Huang and Wang, 2017). Recently, GRASP55 and GRASP65 single knockout mice have been reported, with only limited defects in Golgi structure and function (Veenendaal et al., 2014; Chiritoiu et al., 2019). One possibility is that the knockout effect of one GRASP may be compensated by the redundancy of the other GRASP protein. It has been demonstrated that when one GRASP is depleted in cells, the level of the other GRASP protein may increase to compensate for the knockout effect (Bekier et al., 2017). GRASP55 and GRASP65 double knockout mice have not been reported so far.

## Effects of Golgi Destruction on Cell Attachment, Migration, and Growth

The effect of Golgi unstacking induced by GRASP depletion on cellular activities such as cell attachment, migration, and growth have recently been investigated. GRASP KD or KO in HeLa cells reduces cells adhesion to fibronectin-coated dishes (Ahat et al., 2019). GRASP depletion also reduces cell migration in HeLa and MDA-MB-231 cells. While the effect was significant when a single GRASP was depleted, it was more robust when both GRASPs were removed. Because cell attachment and migration are mediated by cell adhesion molecules, in particular integrins, the level of a variety of integrins in GRASP KO cells was assessed. Among the 10 integrin subunits tested, which can form 8 different heterodimers, α5β1 integrins, the major and well-characterized integrin complex in HeLa, and MDA-MB-231 cells that utilizes fibronectin as its ligand (Mierke et al., 2011), exhibited the most robust reduction upon GRASP depletion. Further analysis showed that GRASP depletion reduces α5β1 integrin levels not only in the cell, but also at the cell surface, providing a reasonable explanation how GRASP depletion reduces cell attachment, and migration (Ahat et al., 2019). Consistently, exogenous expression of α5β1 integrins rescues the attachment and migration defects in GRASP-depleted cells. The effects of GRASP depletion on α5β1 integrins are specific for Golgi unstacking, as disruption of the Golgi ribbon by knocking down Golgin-84 or by nocodazole treatment, or destroying the Golgi structure by brefeldin A treatment, does not reduce integrin levels in cells (Xiang et al., 2013).

There are three possibilities to reduce the α5β1 integrin protein levels in cells by GRASP depletion: decreased synthesis, accelerated degradation, or both. As Golgi structural defects caused by GRASP-depletion may impair protein glycosylation, which plays an important role in protein stability (Shental-Bechor and Levy, 2008), it was initially speculated that Golgi unstacking may impair α5β1 glycosylation and thus reduces their stability. However, the results demonstrated that the reduction of α5β1 integrin is due to decreased protein synthesis rather than increased degradation (Ahat et al., 2019). Interestingly, GRASP-depletion significantly increases total protein synthesis and accelerates cell proliferation (Ahat et al., 2019), consistent with the previous report that GRASP65 depletion accelerates cell cycle progression (Tang et al., 2010). How GRASP depletion selectively decreases the synthesis of α5β1 integrin while increasing overall protein synthesis remains a mystery. One possibility is that the overall protein synthesis is increased due to accelerated cell proliferation and enhanced protein trafficking. In addition, it has been proposed that the Golgi functions as a signal hub, as many signaling molecules are docked on the Golgi membranes and respond to different cellular stresses (Mayinger, 2011; Makhoul et al., 2018). So it is also possible that α5β1 integrin synthesis is regulated by signaling pathways on the Golgi in response to Golgi structural changes caused by GRASP depletion.

## New Discoveries on Grasp55 and Grasp65 Interacting Proteins

Recently, some new discoveries have been made on the known interacting partners of GRASPs. Golgin-45 is one of the earliest identified GRASP55-binding proteins, which is involved in vesicle tethering and Golgi structure regulation (Short et al., 2001; Lee et al., 2014). The structural basis of GRASP55 interaction with Golgin-45 has recently been revealed. The last C-terminal residues of Golgin-45, QGELIAL, insert into the canonical PDZ-peptide binding pocket in the PDZ1 domain of GRASP55, while the upstream residues of the C-terminal sequence of Golgin-45, TRYENITFNCCNHC, interacts with both PDZ domains by inserting into the cleft between them. Furthermore, the C-terminus of Golgin-45 also binds the PDZ2 domains of the two neighboring GRASP55 molecules, which enhances GRASP55 oligomerization. This is thought to play an important role in Golgi stacking. The third interaction site between Golgin-45 and GRASP55 is a unique zinc finger-like structure formed between Cys^393^/Cys^396^ of Golgin-45 and His^18^ (β1)/Cys^103^ (β2) of GRASP55 (Zhao et al., 2017). Similar to the Golgin-45 and GRASP55 interaction, GM130 interacts with GRASP65 via its C-terminal KITVI sequence that binds PDZ1, and via the IPFFY sequence that interacts with both PDZ domains by inserting into the hydrophobic cleft between them. But unlike the Golgin-45 and GRASP55 interaction, GRASP65 undergoes conformational change on PDZ domain upon GM130 interaction but does not form a zinc-finger structure on GM130-GRASP65 interaction (Hu et al., 2015; Zhao et al., 2017).

In the past several years, a number of new GRASP binding proteins have been identified (Figure 1 and Table 1). Two interacting proteins have recently been discovered for GRASP65, the actin elongation factor Mena (mammalian enabled homolog), and the Hsc70 (heat shock cognate 71 kDa protein) co-chaperone DjA1 (DnaJ homolog subfamily A member 1). Mena is recruited to the Golgi membranes by GRASP65 to facilitate actin polymerization and GRASP65 oligomerization, and thus functions as a bridging protein in Golgi ribbon linking (Tang et al., 2016). DjA1 binds to GRASP65 and promotes GRASP65 oligomerization in a Hsc70-independent manner (Li et al., 2019b).

Most recently, several novel GRASP55 binding partners have been identified that are related to the newly discovered function of GRASP55 in autophagy. GRASP55 not only facilitates autophagosome-lysosome fusion via the interactions with LC3 on autophagosomes and LAMP2 on lysosomes (Zhang and Wang, 2018a,b; Zhang et al., 2018), but also directly binds Beclin-1 (BECN1) and UVRAG to facilitate the assembly and membrane association of the phosphatidylinositol 3-kinase (PtdIns3K, or PI3K) complex (Zhang et al., 2019), and therefore plays an important role in autophagosome maturation during both glucose depletion and amino acid starvation. Moreover, GRASP55 binds cystic fibrosis transmembrane conductance regulator (CFTR) and transforming growth factor beta 1 (TGFβ1) to facilitate their unconventional secretion (Gee et al., 2011; Nüchel et al., 2018). These new findings reveal novel roles for GRASPs in cellular activities outside of the Golgi. While some of these findings have been recently reviewed elsewhere (Li et al., 2019a), several key advances are discussed below in detail.

## Grasp55 Regulates Autophagosome-Lysosome Fusion

How the Golgi copes with different stresses and whether there is a Golgi stress sensor have not been systematically studied. In an effort to explore how the Golgi responds to energy deprivation, a number of Golgi proteins were examined for O-GlcNAcylation, a cytosolic glycosylation that serves as an energy sensor to regulate cellular pathways (Zhang et al., 2018). In this study, Zhang et al. discovered that GRASP55, but not other Golgi matrix proteins examined, including GRASP65, GM130 and Golgin-45, is O-GlcNAcylated under growth condition. Upon glucose starvation, GRASP55 is de-O-GlcNAcylated and forms puncta outside of the Golgi area. Since glucose starvation induces autophagy, the function of GRASP55 in autophagy was then tested. Indeed, depletion of GRASP55, but not GRASP65, increased the number of autophagosomes but decreased the autophagic flux, indicating a defect in autophagosome-lysosome fusion.

How does a Golgi protein like GRASP55 regulate autophagy? The study by Zhang et al. (2018) revealed that GRASP55 de-O-GlcNAcylation upon glucose deprivation allows some of the GRASP55 molecules to colocalize with autophagosomes. GRASP55 targeting to autophagosomes is regulated by O-GlcNAcylation, as it is enhanced by mutating the O-GlcNAcylation sites on GRASP55 as well as by glucose starvation which reduces GRASP55 O-GlcNAcylation. Further biochemical studies demonstrate that GRASP55 interacts with LC3-II on autophagosomes, and this interaction is enhanced by GRASP55 de-O-GlcNAcylation. In addition, GRASP55 also interacts with LAMP2 on lysosomes. These together suggest a possibility that GRASP55 may function as a bridging protein to facilitate LC3-LAMP2 interaction as well as autophagosome-lysosome fusion. Indeed, this possibility was subsequently confirmed using *in vivo* and *in vitro* approaches (Figure 2B; Zhang et al., 2018). In cells, GRASP55 depletion reduces LC3 and LAMP2 colocalization as well as autophagosome-lysosome fusion. *In vitro*, GRASP55 facilitates autophagosome-lysosome fusion in an *in vitro* fusion assay. Furthermore, the addition of recombinant GRASP55 enhances LC3 and LAMP2 co-immunoprecipitation from cell lysates (Zhang et al., 2018). Thus, like in Golgi stacking, GRASP55 oligomers serve as membrane tethers to facilitate autophagosome-lysosome fusion.

The role of GRASP55 in autophagosome-lysosome fusion is not limited to glucose starvation, but also in amino acid starvation. Upon amino acid starvation, GRASP55 not only physically interacts with LC3 and LAMP2, but also regulates the formation of the PI3K UVRAG complex that is known to facilitate autophagosome-lysosome fusion (Zhang et al., 2019). Here, GRASP55 directly interacts with Beclin-1, induces the UVRAG PI3K complex formation and increases its membrane association. These reports identified GRASP55 as a specific energy and nutrient sensor on the Golgi to regulate autophagy.

Since GRASP55 still appears on autophagosomes in the presence of the protein synthesis inhibitor cycloheximide, it is speculated that GRASP55 is targeted to autophagosomes from an existing pool upon autophagy induction (Zhang et al., 2018). One remaining interesting question concerns how GRASP55 is targeted to autophagosomes. GRASP55 is unlikely translocated to the autophagosomes with the entire Golgi as cargo for autophagic degradation, since no other Golgi markers are found in the newly formed autophagosomes, and GRASP55 is localized on the outer membrane of autophagosomes instead of the lumen (Zhang et al., 2018, 2019). One possibility is through vesicular transport, similar to the transmembrane protein Atg9 that is normally enriched in the Golgi and is translocated to autophagosomes upon autophagy induction. However, unlike Atg9, GRASP55 does not have a transmembrane domain, and how the translocation occurs selectively on de-O-GlcNAcylated GRASP55 remains unknown. Alternatively, it is possible that a small pool of GRASP55 constantly shuttles between the Golgi, cytosol, and autophagosomes; and the equilibrium is regulated by GRASP55 O-GlcNAcylation. Nevertheless, how GRASP55 is targeted to autophagosomes under stress conditions requires further investigation.

While the discovery of GRASP55 as a membrane tether in autophagosome-lysosome fusion is exciting, it is unknown how GRASP55 interplays with other known tethering proteins, in particular Rab7 and the HOPS complex, as well as the STX17-SNAP29-VAMP7/8 SNARE complex that mediates the fusion (Jager et al., 2004; Itakura et al., 2012; Jiang et al., 2014). It is possible that these proteins function sequentially during autophagosome-lysosome fusion. Alternatively, GRASP55 may function as an independent mechanism in autophagosome-lysosome fusion.

## Grasp55/65 and Unconventional Secretion

It was long believed that only proteins with canonical ER signal peptides at the N-terminus can be secreted out of the cell. Since the discovery of the Golgi-independent unconventional secretion of the cytokine interleukin 1 beta (IL-1β) (Muesch et al., 1990), more proteins without ER signal sequences (leaderless proteins) and some integral membrane proteins have been reported to be transported or secreted in a Golgi-independent manner (Kinseth et al., 2007; Schotman et al., 2008; Gee et al., 2011). Collectively, the non-canonical Golgi-independent secretion is referred to as unconventional protein secretion (UPS). Interestingly, although UPS itself is Golgi-independent, it requires two important Golgi proteins, GRASP55 and GRASP65, in mammalian cells and their homologs in other model organisms. Whether unconventional secretion is a by-product of loss of Golgi functions, or vice-versa, has not been ruled out due to the lack of molecular tools to manipulate Golgi structure, and function without affecting GRASPs. In a previous review (Rabouille, 2017), unconventional secretion is generally classified into four categories, type I is direct translocation of cargo molecules across the plasma membrane via pore formation, type II is unconventional secretion through ATP-binding cassette (ABC) transporters, type III is vesicle-mediated secretion of cytosolic proteins, and type IV is the Golgi bypassing transportation of integral membrane proteins. GRASPs and their homologs have been reported to play a role in type III and type IV UPS, which is the focus of this review in terms of unconventional secretion.

### GRASPs and Unconventional Trafficking of Integral Membrane Proteins

#### CFTR

The Phe508 deletion from CFTR results in the inhibition of its trafficking to the plasma membrane and ER-associated degradation. It has been shown that GRASP55 and GRASP65 are required for the unconventional secretion of both WT and DeltaF508 CFTR when ER-to-Golgi trafficking is inhibited by expressing a dominant negative Sar1 or Arf1, or by inhibiting vesicle fusion via overexpression of syntaxin 5 (Gee et al., 2011). GRASP55 is phosphorylated at Ser441 during certain ER stresses, monomerized, and located near the ER to regulate unconventional secretion of CFTR, which requires CFTR-GRASP55 interaction (Figure 2C; Kim et al., 2016; Gee et al., 2018). Similar to GRASP55, GRASP65 overexpression rescued the secretion defect of mutant CFTR, although GRASP65 was not extensively tested in the same studies.

One contradiction in these reports is that overexpression of the GRASP55-G2A mutant inhibits CFTR secretion, whereas expression of N-terminally tagged GRASP55, which has been shown to abolish its membrane targeting similar to the GRASP-G2A mutant, increases CFTR secretion (Gee et al., 2011; Heinrich et al., 2014; Kim et al., 2016). Considering that GRASP55 regulates CFTR secretion by direct binding, GRASP55 may serve as a cytosolic chaperone to regulate CFTR sequestration by autophagosomes or by multivesicular bodies (MVBs) (Noh et al., 2018). Here, GRASP55 phosphorylation and re-localization in response to ER stress is necessary for CFTR secretion. A recent report showed that GRASP55 regulates the IRE1 ER unfolded protein response (UPR) pathway (Chiritoiu et al., 2019). It is unclear why the *medial/trans*-Golgi protein GRASP55, but not the *cis*-Golgi protein GRASP65, is the main player in regulating this activity as GRASP65 is localized on the Golgi compartments closer to the ER.

#### Integrin

dGRASP, the *Drosophila* homolog of GRASP, regulates the Golgi bypassing, non-canonical trafficking of the alpha subunit of integrin during *Drosophila* wing development (Schotman et al., 2008). Unlike normal situations in which dGRASP is localized on the Golgi, during wing development, dGRASP localizes near the plasma membrane to regulate non-canonical secretion of alpha-integrin. It was proposed that dGRASP regulates integrin secretion via facilitating the fusion of integrin-containing vesicles with the plasma membrane (Schotman et al., 2008). Alternatively, GRASP depletion may cause mis-sorting of an unknown, plasma membrane destined vesicle fusion protein (i.e., SNARE), which subsequently affects the non-canonical secretion of integrin. Unlike CFTR, it is unclear if dGRASP binding is required for integrin secretion. Considering that GRASP55 translocates to the ER in CFTR secretion while dGRASP translocates to near the plasma membrane area in integrin secretion, GRASP may regulate the secretion of these two transmembrane cargoes through different mechanisms.

### GRASP55 and Endosome/Autophagosome-Dependent Unconventional Secretion of Cytosolic Proteins

#### IL-1β

Interleukin 1 beta, the major cytosolic regulator of inflammation, was one of the first identified cargoes of UPS (Muesch et al., 1990). IL-1β and another cytokine, TGFβ1, are secreted via a non-canonical secretory pathway distinct from the conventional ER-Golgi pathway (Nüchel et al., 2018). IL-1β is exported either through vesicle mediated secretion or via Gasdermin-D-mediated pore formation at the plasma membrane (Kayagaki et al., 2015). More recently, it was revealed that IL-1β is first restrained in the intermembrane space of autophagosomes and then secreted by the fusion of autophagosomes with the plasma membrane (Figure 2D). This fusion process is mediated by SNAREs including Sec22b on autophagosomes, syntaxin 3 and syntaxin 4 on the plasma membrane, and SNAP-23 and SNAP-29 from the cytosol (Dupont et al., 2011; Kimura et al., 2017). Most recently, a question was raised on whether autophagy is indeed involved in IL-1β secretion (Chiritoiu et al., 2019).

Interestingly, depletion of GRASP55 or GRASP65 reduces IL-1β secretion (Zhang et al., 2015; Figure 2D). GRASP55 may regulate the secretion of IL-1β through its role in autophagy or by modulating the IREα/XBP-1 UPR pathway (Dupont et al., 2011; Zhang and Wang, 2018a; Chiritoiu et al., 2019; van Ziel et al., 2019). Considering the function of GRASP55 in autophagosome-lysosome fusion, it is reasonable to speculate that GRASP55 may also be involved in autophagosome-plasma membrane fusion in this scenario.

#### AcbA/Acb1

GRASP65 and GRASP55 were originally identified in mammalian cells to regulate Golgi stacking and ribbon linking. Kinseth et al. (2007) provided the first evidence that GRASP regulates unconventional secretion in *Dictyostelium*. This unexpected finding came from the observation that knockout of GrpA, the GRASP homolog in *Dictyostelium*, results in a secretion defect of AcbA, a protein that is processed in the extracellular environment to produce the spore differentiation factor-2 (SDF 2) (Kinseth et al., 2007; Levi and Glick, 2007). Later, it was reported that Acb1, the budding yeast homolog of ACBP, is also unconventionally secreted, which depends on Grh1 (the GRASP homolog in the budding yeast), core Atg genes, ESCRT machinery and SNAREs, but is independent of COPII coated vesicles (Figure 2D; Duran et al., 2010; Manjithaya et al., 2010).

Although GRASP is required for Acb1 secretion, it is not clear at which step GRASP is involved. It seems that the vesicles carrying Acb1 are distinct from autophagosomes because GFP-Grh1 colocalizes with neither the autophagosome marker Atg8 nor the phagophore marker Ape1 (Bruns et al., 2011). Instead, a novel membrane-bound compartment called CUPS (compartment for UPS) is required for Acb1 secretion. However, the formation and identity of this membrane structure are largely unknown (Ye, 2018).

## Outlook

It is clear that GRASP65 and GRASP55 have both Golgi-dependent and Golgi-independent functions. On the Golgi, GRASP *trans*-oligomers are the primary machineries for Golgi stack formation. The recent finding of reduced cell adhesion and migration under GRASP depletion strengthens the essential role of Golgi stacking in protein trafficking, modification, and signaling.

Under certain stress conditions or at certain stages of development, GRASPs also function outside of the Golgi, likely as membrane tethers as within the Golgi stacks. The findings are interesting, but several outstanding questions remain. For example, how is GRASP55 targeted to different locations outside of the Golgi? Do GRASPs function as tethers in the regulation of unconventional secretion? The recent findings of GRASP55 in autophagy may help address these questions. Furthermore, the role of GRASPs in unconventional secretion has been confirmed in a variety of systems, but not all types of unconventional secretion require GRASPs. For example, unconventional secretion of a misfolding-associated protein secretion (MAPS) cargo GFP1-10 and a cilia transmembrane protein Peripherin/rds is reported to be GRASP-independent (Tian et al., 2014; Lee et al., 2016). This indicates a heterogeneity of unconventional secretion and the specificity of GRASPs in the regulation of unconventional secretion of certain substrates. Future studies by comparing different cargo molecules may help understand the exact roles of GRASPs in unconventional secretion.

It is clear that GRASP proteins play important roles in unconventional secretion, but the underlying mechanism remains largely unknown. Both unconventional secretion of certain cargoes and autophagy are augmented under stress conditions. The newly uncovered roles of GRASP55 as an energy sensor in the Golgi and a membrane tether in autophagy indicate that it may serve as a stress sensor and an effector in stress response; and these roles may be linked to unconventional secretion of certain cargo molecules. GRASP55 may coordinate Golgi-dependent and Golgi-independent trafficking pathways in the cell under different conditions. In addition to autophagy, the emerging role of GRASP55 in the regulation of ER stress and UPR indicates that GRASP55 may affect unconventional secretion through UPR (Chiritoiu et al., 2019; van Ziel et al., 2019). Systematic studies on Golgi response to different stress stimuli and identification of novel GRASP interacting proteins under normal and stress conditions may shed light on the mechanism of GRASP55 and GRASP65 in Golgi-dependent and Golgi-independent functions.

## Author Contributions

All authors contributed to the design, conception and manuscript preparation. All authors approved the publication of this study.

## Conflict of Interest Statement

The authors declare that the research was conducted in the absence of any commercial or financial relationships that could be construed as a potential conflict of interest.
